# Phosphorylation Signaling in APP Processing in Alzheimer’s Disease

**DOI:** 10.3390/ijms21010209

**Published:** 2019-12-27

**Authors:** Tao Zhang, Dongmei Chen, Tae Ho Lee

**Affiliations:** Fujian Key Laboratory for Translational Research in Cancer and Neurodegenerative Diseases, Institute for Translational Medicine, School of Basic Medical Sciences, Fujian Medical University, Fuzhou 350122, China; zhangtaond1991@163.com (T.Z.); dmchen88@126.com (D.C.)

**Keywords:** Alzheimer’s disease, APP processing, phosphorylation, amyloid-β, kinase

## Abstract

The abnormal accumulation of amyloid-β (Aβ) in the central nervous system is a hallmark of Alzheimer’s disease (AD). The regulation of the processing of the single- transmembrane amyloid precursor protein (APP) plays an important role in the generation of Aβ in the brain. The phosphorylation of APP and key enzymes involved in the proteolytic processing of APP has been demonstrated to be critical for modulating the generation of Aβ by either altering the subcellular localization of APP or changing the enzymatic activities of the secretases responsible for APP processing. In addition, the phosphorylation may also have an impact on the physiological function of these proteins. In this review, we summarize the kinases and signaling pathways that may participate in regulating the phosphorylation of APP and secretases and how this further affects the function and processing of APP and Aβ pathology. We also discuss the potential of approaches that modulate these phosphorylation-signaling pathways or kinases as interventions for AD pathology.

## 1. Introduction

Current epidemiological studies estimate that there are approximately 45 million people suffering from dementia [1]. With the global trend of aging, this number is predicted to triple by 2050 [2]. Alzheimer’s disease (AD) is a neurodegenerative disease characterized by a progressive decline in cognition and memory abilities in the elderly. It has been identified as the leading cause of dementia, accounting for more than 60% of all dementia cases worldwide [3]. About 95% of AD patients develop the disease without a definitive cause, which is termed as sporadic AD [4]. The remaining AD cases, known as familial AD, are thought to be caused by mutations in genes encoding three important proteins associated with AD, namely, amyloid precursor protein (APP), presenilin 1 (PS1), and presenilin 2 (PS2) [5]. Familial AD patients usually develop disease symptoms much earlier than sporadic AD patients [5]. There are two typical pathological hallmarks in AD: the accumulation of amyloid plaques consisting of amyloid-β (Aβ) proteins in the brain parenchyma of AD patients and the formation of neurofibrillary tangles composed of hyperphosphorylated tau filaments [6]. Additional pathological changes in AD include the presence of chronic neuroinflammation, gradual neuronal loss and synaptic dysfunction, and impairments in the blood–brain barrier (BBB) [7]. Age is the biggest nongenetic risk factor for AD, as revealed by the exponential increase in the incidence of AD with the advancement of age [2]. Although the exact etiology of sporadic AD remains largely undefined, it is increasingly recognized that both genetic and environmental factors play important roles in the progression of AD. For instance, Apolipoprotein E (ApoE) is responsible for lipid transportation and injury repair in the brain. *ApoE ε4* has been suggested to be a major genetic risk factor for sporadic AD [8]. Individuals carrying two copies of *ApoE ε4* may have a 5–8-fold increase in the risk of developing AD [9]. Mutations in *APP*, *PS1* and *PS2* could cause autosomal dominant familial AD, which usually causes disease symptoms before the age of 65 [9]. Genome-wide association studies (GWAS) have been applied to identify single nucleotide polymorphisms (SNPs) of genes that may be involved in AD, especially late-onset AD. For example, SNPs in genes regulating lipid homeostasis and immune function (e.g., *phosphatidylinositol binding clathrin assembly protein* (*PICALM*) for lipid metabolism and *complement receptor type 1* (*CR1*) and *triggering receptor expressed on myeloid cells 2* (*TREM2*) for immune regulation) have also been associated with the risk of developing AD [1,10]. To date, there are only four symptomatic treatments available for the management of AD-induced dementia. Three of these drugs are cholinesterase inhibitors (ChEIs), which suppress the activity of cholinesterases in synaptic clefts and increase the content of acetylcholine in the brain, thereby improving memory and cognition [11]. The fourth drug, memantine, is an N-methyl-D-aspartate (NMDA) receptor antagonist that reduces calcium overload in neurons and prevents subsequent neurotoxicity [12].

The phosphorylation of proteins is a fundamental posttranslational modification whereby target molecules are covalently modified at serine, threonine or tyrosine residues by the addition of phosphate groups [13]. The regulation of protein phosphorylation is based on crosstalk between protein kinases and protein phosphatases via complex signaling pathways. Most of the phosphorylation occurs on serine residues of a protein. The phosphorylation of threonine and tyrosine residues is less common than that of serine residues [13]. The phosphorylation of a protein introduces negatively charged phosphate groups on the protein, leading to changes in the conformation and functional activities of the protein [14]. For example, the phosphorylation and dephosphorylation of tau proteins are essential for the polymerization of tubulin and the stabilization of microtubules [15]. The dysregulation of tau phosphorylation may lead to the aggregation of tau and the formation of paired helical filaments (PHFs) [15]. The protein phosphorylation signaling pathway is also closely involved in a variety of cellular events and cell fates, indicating the crucial role of phosphorylation signaling in both physiological and pathological conditions. Phosphorylation signaling in AD is mostly focused on tau proteins, due to their abundant phosphorylation sites and kinases, while much less is known about the phosphorylation of the APP and its impact on the function and processing of APP. The present review also discusses the kinases and phosphorylation signaling during the metabolism of APP.

## 2. APP Processing

**Figure 1:**
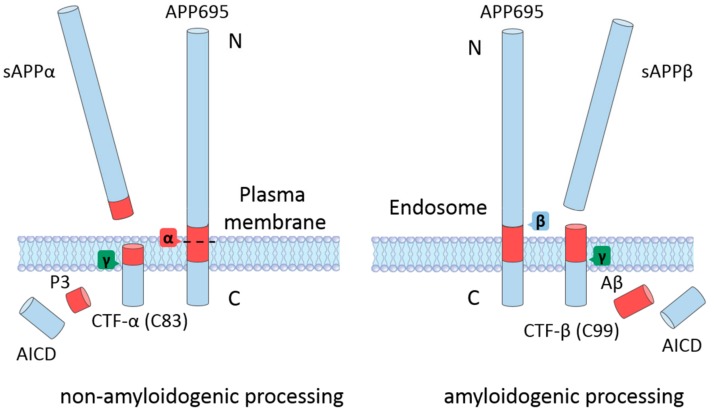
The proteolytic cleavage of the amyloid precursor protein (APP) by the non-amyloidogenic (**left**) and amyloidogenic (**right**) processing pathways. In the non-amyloidogenic pathway, mature APP anchored to the plasma membrane is processed by α-secretase within the amyloid-β (Aβ) region (shown in red) and releases soluble APPα (sAPPα) and the C-terminal fragment-α (CTF-α). The CTF-α is further processed by γ-secretase to generate P3 and the APP intracellular domain (AICD). In the amyloidogenic pathway, which preferentially occurs in acidic environments such as endosomes, reinternalized APP is consecutively cleaved by β- and γ-secretase to produce soluble APPβ (sAPPβ), Aβ, and AICD. The C-terminal fragment-β (CTF-β) is an intermediate product of the β-secretase cleavage.

APP is a type-I single transmembrane protein with a long extracellular domain and a short cytoplasmic domain [16]. The *APP* gene is located on chromosome 21. The alternative splicing of the *APP* gene produces three isoforms: APP695, APP751, and APP770 (the numbers indicate the number of amino acid residues). The extracellular domain of APP contains two independently folded E1 and E2 subdomains and a juxtamembrane domain [17]. The transmembrane domain (TMD) is relatively hydrophobic and is buried in the cell membrane. The remaining amino acid residues are located in the cytoplasm and form the APP intracellular domain (AICD). APP695 is predominantly expressed in neurons in the central nervous system (CNS), while the other two isoforms can be found in most tissues [18]. The physiological function of APP is not yet fully understood. APP might have certain roles during neuronal differentiation and development, which are mediated either by the intact form or by the processing products [16]. Upon generation in the endoplasmic reticulum (ER), immature APP undergoes posttranslational modifications and matures through the constitutive secretory pathway [19]. When APP reaches the cell surface, it may be further cleaved by different processing pathways, depending on its trafficking and localization (Figure 1) [19]. It has been shown that APP residing at the cell surface is mainly cleaved by α-secretases that are also enriched at the cell membrane [20]. APP may also be internalized again to the endosome and encounter β-secretases, thereby initiating the amyloidogenic processing pathway [20].

### 2.1. Non-Amyloidogenic Processing of APP

The cleavage of APP by α-secretase does not produce Aβ proteins in neurons because the cleavage site of α-secretase is located within the Aβ domain (the peptide bond formed by Lys16 and Leu17 in the numbering of Aβ40) (Figure 1, left panel) [21]. This processing pathway first generates a long soluble extracellular fragment (soluble APPα (sAPPα)) and a C-terminal fragment (CTF-α (C83)). The CTF-α can further be cleaved by γ-secretase to produce the P3 fragment and the AICD [17]. Several zinc metalloproteinases in the a disintegrin and metalloproteinases (ADAM) family can act as α-secretases for the non-amyloidogenic processing of APP [22]. Although the ADAM family contains a group of enzymes, including ADAM9, ADAM10, and ADAM17, it has been shown that ADAM10 is the principal α-secretase that mediates APP processing in neurons [22,23]. ADAM17 can also carry out the α-secretase cleavage of APP [24]. The plasma membrane localization of α-secretases facilitates the non-amyloidogenic APP processing which also predominantly occurs at the cell surface.

Interestingly, the non-amyloidogenic processing pathway not only avoids the generation of aggregation prone Aβ but also leads to the formation of neuroprotective sAPPα, as indicated in a number of studies [25]. For example, sAPPα is able to protect the brain against traumatic brain injury (TBI) and transient ischemia-induced neuronal death and axonal damage [26]. Obregon et al. showed that sAPPα can directly interact with β-secretase, leading to a reduction in the generation of Aβ [27]. It may also protect neurons against Aβ oligomer-induced dendritic spine loss and tau phosphorylation [28]. The protective effect of sAPPα highlights the promising application of the modulation of AD pathology via the promotion of the non-amyloidogenic processing of APP.

### 2.2. Amyloidogenic Processing of APP

After maturation, APP is trafficked to the acidic endosomal environment through endocytosis, whereby the protein encounters β- and γ-secretase and undergoes successive cleavage to produce Aβ (Figure 1, right panel) [29]. The first step in the generation of Aβ is the β-secretase cleavage of APP between Met596 and Asp597 by the β-site APP-cleaving enzyme 1 (BACE1), which results in the generation of a soluble APP N-terminal fragment (sAPPβ) and a C-terminal fragment (CTF-β (C99)) [18]. Following the cleavage of the γ-secretase complex at the transmembrane domain of APP, the Aβ protein and AICD are formed and released into the cytoplasm [18]. BACE1 is the only β-secretase that can process APP; therefore, inhibiting BACE1 could significantly reduce the generation of Aβ in the brain. γ-Secretase is a protease complex with four subunits. The PS1 or PS2 subunit is responsible for the catalytic ability of γ-secretase, while the other three subunits (nicastrin, anterior pharynx-defective 1, and the PS enhancer) mainly participate in substrate recognition, complex assembly, and the autocatalytic cleavage of PS [30,31,32]. The cleavage of γ-secretase is not confined at a single site; instead, it can take place between the amino acid residues 37 to 43 of the Aβ domain [19]. This variation is directly associated with the proteolytic product of the amyloidogenic processing of APP. Aβ40 and Aβ42 are the dominant products of the amyloidogenic processing pathway, and other minor cleavage products include Aβ38, Aβ39, and Aβ43 [33]. Upon generation, Aβ monomers may associate together and form aggregates of different sizes, morphologies, and toxicities, ultimately leading to amyloid deposition in the brain.

## 3. Phosphorylation Signaling in APP Processing

According to the amyloid cascade hypothesis, dyshomeostasis in the generation and clearance of Aβ increases the content of Aβ species in the brain, subsequently initiating the oligomerization and fibrillation of Aβ [34]. The aggregation of Aβ, especially the presence of Aβ oligomers, may further induce neuroinflammation, oxidative damage, and calcium dyshomeostasis [35]. Recent studies have also noted that Aβ aggregation is able to exacerbate tau pathology by promoting the phosphorylation and aggregation of tau proteins [36]. Consequently, the amyloid and tau pathologies cause irreversible synaptic damage and neuronal dysfunction in brain regions responsible for learning and memory. Considering the central role of Aβ misfolding and aggregation in the pathogenesis of AD, understanding the regulation of APP processing would be of great importance to clarify the etiology of AD, as well as to identify novel therapeutic targets for disease intervention. In the following sections, we discuss the phosphorylation signaling of all proteins involved in APP processing pathways and how the signaling influences APP metabolism and Aβ formation.

### 3.1. APP Phosphorylation

**Figure 2:**
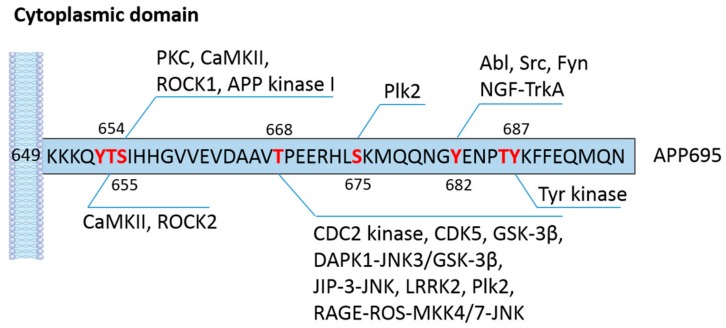
Phosphorylation sites of APP695 in the cytoplasmic domain and the reported kinases/signaling pathways. Eight phosphorylation sites (marked in red) have been reported in the cytoplasmic domain of APP695. Kinases and signaling pathways that are able to phosphorylate these amino acid residues according to the literature are summarized in the scheme. See Table 1 for full list of abbreviations.

APP has several phosphorylation sites in both the cytoplasmic and extracellular domains (Table 1). The phosphorylation of APP at its short cytoplasmic domain has been implicated not only in cell and animal models but also in a plethora of clinical studies. There are eight potential phosphorylation sites in the cytoplasmic domain of APP: threonine (T) residues at 654, 668, and 686; serine (S) residues at 655 and 675; and tyrosine (Y) residues at 653, 682, and 687 (all in APP695 isoform numbering) (Figure 2) [37]. It has been shown that seven of the eight phosphorylation sites (except T654) are observed in brains of AD patients [38], suggesting that APP phosphorylation may have a significant impact on the physiological function and processing of APP in the CNS. The phosphorylation of APP at T668 has attracted tremendous interest because of its prevalence in AD and its pronounced effect on APP cleavage [39]. Using synthetic APP fragments as substrates, Suzuki and colleagues reported that APP can be phosphorylated by p34cdc2 protein kinase (CDC2 kinase) at T668 in vitro. Furthermore, they also found that cellular APP was phosphorylated by CDC2 kinase in a cell cycle-dependent manner. The elevated phosphorylation of APP at T668 in the G2/M phase increases the content of immature APP and C-terminal fragments while reducing the level of secreted APP products [40]. It should be noted that the precise metabolic processing of APP and the exact enzymes involved in this process were not fully resolved at the time of the study, which made it difficult to characterize how and why the metabolism of APP is changed by phosphorylation. Afterwards, more serine/threonine kinases were found to be capable of phosphorylating the T668 residue, including glycogen synthesis kinase-3β (GSK-3β) [41,42], cyclin-dependent kinase 5 (CDK5) [43,44], extracellular signal-regulated kinase (ERK) 1 [45], and c-Jun N-terminal kinase (JNK) [46,47,48]. Lee et al. demonstrated that APP phosphorylation at T668 is upregulated in the hippocampus of AD patients [38]. Additionally, phosphorylated APP colocalized with BACE1 in enlarged endosomes in hippocampal neurons. The inhibition of APP phosphorylation at T668 significantly decreases the generation of Aβ, implicating a direct association between APP phosphorylation and BACE1 mediated cleavage [38]. In addition to its presence in Alzheimer’s disease, APP phosphorylation has also been found to occur during cellular stress and mitosis and in differentiated neurons [49]. The phosphorylation of APP under various conditions is likely caused by distinct signaling pathways and may induce different consequences on the subcellular localization of APP. In differentiated neurons, APP is mainly phosphorylated by the GSK3β/JIP-3/JNK pathway and is transported anterogradely to distal neurites [49,50]. The phosphorylation of APP in degenerating neurons depends largely on the CDK5 pathway, and the products are mainly enriched in endosomes in the cell body [49]. In a cellular model mimicking AD, Colombo et al. observed that JNK activation enhances the phosphorylation of APP at the T668 site and favors the amyloidogenic cleavage of APP. The inhibition of JNK via D-JNKI1 peptides induces APP degradation and shifts the processing of APP toward the non-amyloidogenic pathway, therefore lowering the content of soluble Aβ oligomers in neurons [51]. Similar findings showing that the functional loss of JNK signaling by modulating mitogen-activated protein kinase (MAPK) kinase 4/7 (MKK4/7) significantly reduces the generation of Aβ and amyloid plaque formation in an AD mouse model were reported [52]. In support of this, the deactivation of JNK3 protects neurons against Aβ aggregation-induced metabolic stress and the resulting JNK3 mediated T668 phosphorylation of APP, leading to a dramatic reduction in Aβ formation and plaque burden and significant improvement in neuronal functions [53]. Death-associated protein kinase 1 (DAPK1) is a calcium/calmodulin-dependent serine/threonine kinase that is highly expressed in the brains of AD patients. Our previous study discovered a close connection between DAPK1 activation and the T668 phosphorylation of APP [54]. Kim et al. observed a positive correlation between DAPK1 levels and APP phosphorylation levels in the brains of AD patients [54]. In addition, the activation of JNK3 and GSK-3β is required for DAPK1-induced T668 phosphorylation in neurons [54]. In line with these results, the knockdown of DAPK1 or inhibition of its enzymatic activity remarkably decreases the secretion of Aβ [54]. These discoveries further confirm the important role of T668 phosphorylation in regulating APP processing and Aβ biosynthesis.

Interestingly, APP phosphorylation at T668 seems to play a role in the pathogenesis of Parkinson’s disease (PD). Chen and co-workers proved that leucine-rich repeat kinase 2 (LRRK2) is capable of interacting with and phosphorylating APP at T668 residues. Following cleavage, the phosphorylated AICD translocates to the cell nucleus with elevated transcriptional activity and exacerbates LRRK2-mediated neurotoxicity and dopaminergic neuronal loss [63]. Most of the reported kinases for T668 are proline-directed protein kinases that are commonly involved in regulating cell proliferation and stress responses. Ramelot et al. discovered that phosphorylation at T668 stabilizes the cis isomer of APP cytoplasmic tails through transient hydrogen bonds [74], which suggests that the prolyl isomerization of the phosphorylated T–P peptide bond might also be altered in response to the phosphorylation process. This conformational change may in turn affect the interaction between APP and other proteins, as well as the trafficking and processing of APP. Peptidyl-prolyl cis/trans isomerase NIMA-interacting 1 (Pin1) has been shown to regulate the conformation and function of phosphorylated tau in AD [75]. Further genetic evidence has revealed that Pin1 expression is irreversibly associated with neuronal loss and neurofibrillary degeneration in AD [76]. Additionally, Pin1 knockout mice manifested gradual cognitive dysfunction and tau pathology [76]. Pastorino et al. discovered that Pin1 can bind to the phosphorylated T668-P motif of APP both in vitro and in vivo. The binding catalyzes the isomerization of phosphorylated APP from cis to trans conformation, thereby promoting the non-amyloidogenic processing of APP and reducing Aβ generation [77,78]. The upregulation of APP T668 phosphorylation could also be a consequence of the deactivation of protein phosphatase (PP) in vivo. AD patients have an increased level of cancerous inhibitor of protein phosphatase 2A (CIP2A) compared with that of age-matched controls [79]. CIP2A inhibits the activity of PP2A and promotes APP T668 phosphorylation, triggering the β-cleavage of APP, Aβ production, and tau hyperphosphorylation [79]. Finally, CIP2A damages synaptic function and causes memory impairments in mouse models [79]. This finding corroborates the essential role of APP phosphorylation in the modulation of Aβ formation and offers new insight into the pathogenesis of AD. In contrast with the evidence highlighting the pivotal role of T668 phosphorylation in modulating APP processing, research on a mouse model carrying the APP T668A mutation showed that the knock-in mice have almost identical levels of APP holoproteins and cleavage products (sAPPα/β, CTF-α/β, and Aβ40/42,) as those found in wild type (WT) mice [80]. There is also no difference with respect to the subcellular distribution of APP proteins between these two mouse models [80]. That the APP T668A knock-in mice show a similar APP processing profile to that of WT mice does not fully contradict the involvement of T668 phosphorylation in regulating Aβ formation under pathological conditions. In addition to the modulation of APP cleavage, the phosphorylation of T668 could also interfere with the interaction between APP and its binding partners. For instance, FE65 is an adaptor protein that can form complexes with the intracellular domain of APP and regulate DNA transcription. FE65 has been found to be tethered to the membrane by its binding to APP, while APP phosphorylation disrupts the membrane tethering and releases free FE65 into the cytoplasm, which further influences the nuclear translocation and signaling of FE65 in neurons [81,82,83]. It should also be mentioned that the T668 phosphorylation occurs not only in full-length APP, but also in C-terminal fragments of APP derived from secretase cleavage [84]. Bukhari et al. found that membrane-tethered C99 fragments can be phosphorylated at T668 by JNK3A. This phosphorylation event seems to reduce the turnover of APP and the formation of the so-called nuclear spheres consisting of FE65, translocated AICD, and other proteins in the cell nucleus [84,85]. One of the physiological functions of APP is to modulate neurite outgrowth in the hippocampus [86,87]. The phosphorylation of APP at T668 is reported to correlate with the neurite outgrowth and the differentiation of PC12 cells [88]. These findings implicate that the T668 phosphorylation of APP might possess diverse functions in the brain.

Although the T668 residue appears to be the most extensively studied phosphorylation site on the APP, the first reported phosphorylation sites were T654 and S655, which were identified by Gandy et al. using synthetic peptides corresponding to the sequence of APP645–661 [55]. The authors identified protein kinase C (PKC) and Ca^2+^/calmodulin-dependent protein kinase II (CaMKII) as the catalytic enzymes of the phosphorylation process [55]. PKC phosphorylates S655, while the CaMKII is able to phosphorylate both threonine and serine residues. Further studies from the same group revealed that mature APP and C-terminal fragments of APP can also be phosphorylated by PKC at S655 [89]. Isohara and colleagues discovered that APP kinase I, which is purified from rat brain tissues, could also phosphorylate the S655 residue of APP [61]. Surprisingly, the activation of PKC or the suppression of protein phosphatases by small molecules is able to increase the secretion of APP into the cerebrospinal fluid (CSF) and decrease the proteolytic cleavage of mature APP [57,58,90,91], while the direct impact of S655 phosphorylation on APP processing remains controversial. The S655 residue lies in the ^653^YTSI^656^ motif, which is involved in the trafficking and sorting of APP [92]. Using fluorescence imaging, Vieira et al. demonstrated that APP phosphorylated at S655 preferentially accumulates in the Golgi apparatus and the plasma membrane, where the α-secretase cleavage of APP is relatively favored [59]. A recent study by Hu et al. illustrated that Rho-associated coiled-coil kinase 1 (ROCK1) directly interacts with APP and phosphorylates the S655 residue [60]. The outcome of this phosphorylation is strengthening of the amyloidogenic processing of APP as it promotes the interaction between APP and BACE1 [60]. ROCK1 inhibition attenuates the accumulation of Aβ and improves AD pathology in AD transgenic mice. It is also reported that ROCK1 is elevated in patients with mild cognitive impairment (MCI) and AD [93]. Henderson et al. demonstrated a mutual regulating cycle between Aβ production and ROCK1 activation [93]. Aβ oligomers can activate ROCK1 without influencing the protein level, which in turn may result in increased generation of Aβ; while inhibiting ROCK1 significantly reduces Aβ production likely by enhancing the degradation of APP [93]. ROCK2 is highly homologous to ROCK1 with respect to the amino acid sequence. ROCK2 is able to phosphorylate T654 according to mass spectrometry [56]. The inhibition of ROCK2 not only suppresses the activity of BACE1 and blocks the T654 phosphorylation of APP but also redistributes BACE1 and APP in subcellular compartments [56]. The generation of Aβ is also dramatically reduced by ROCK2 inhibition, probably due to a combination of both mechanisms [56]. The sorting protein-related receptor with A-type repeats (SorLA or LR11) is an APP-interacting protein which negatively regulates the trafficking of APP to secretases, like BACE1, and the production of Aβ [94]. ROCK2 could phosphorylate the S2206 residue of SorLA and may further affect SorLA-mediated APP trafficking and Aβ production [95].

S675 is another serine residue that has been detected to be phosphorylated in the brains of AD patients, but its function was only recently clarified. Lee et al. reported that Polo-like kinase 2 (Plk2) is able to phosphorylate both T668 and S675 residues [64]. Plk2 is sensitive to neuronal activity and plays a role in regulating synaptic function. The overactivation of neurons, such as that which occurs in the presence of Aβ oligomers, induces the expression of Plk2 at somatodendrites, wherein the kinase directly interacts with the intracellular domain of APP and phosphorylates the T668 and S675 residues. Phosphorylation stimulates the endocytosis of APP and drives the BACE1 mediated cleavage, thereby increasing the production of Aβ in the synapse [64]. By using a phospho-mimicking protein of APP, Menon et al. showed that S675 phosphorylation increases the noncanonical APP processing by meprin β but decreases the α-secretase cleavage at the plasma membrane. This could potentially contribute to AD pathology since APP processing by meprin β leads to aggregation-prone, truncated Aβ species [96].

There are three tyrosine residues (Y653, Y682, and Y687) within the cytoplasmic domain of APP. The Y653 residue has been observed to be critical for the polarized sorting of APP to the cell surface [97]. The most studied tyrosine phosphorylation site present in the human brain is the Y682 residue [98]. It has been demonstrated that the ^682^YENPTY^687^ motif has important effects on the trafficking and metabolism of APP [99,100,101]. Mutations of the Y682 residue cause progressive synaptic loss and cholinergic dysfunction and impair learning and cognitive abilities in mouse models [102,103]. This motif could also interact with proteins containing phosphotyrosine-binding (PTB) domains. The diverse role of Y682 in the CNS suggests that it may be crucial for the physiological function of APP. Y682 was first found to be phosphorylated by two non-receptor tyrosine kinases, Abl and Src, and tropomyosin receptor kinase A (TrkA) is the other tyrosine kinase that has been demonstrated to phosphorylate the Y682 residue of APP [65,66,68]. Nerve growth factor (NGF) is a neurotrophic factor that, upon binding to its receptor TrkA, can stimulate neurogenesis. NGF can trigger Y682 phosphorylation by activating TrkA, and this phosphorylation then facilitates the interaction between TrkA and APP. The complex crosstalk influences both APP processing by modulating the γ-secretase cleavage and the neurotrophic signaling pathway [67,68]. After phosphorylation, the interaction between APP and the PTB domain of the cytoplasmic adapter protein Shc A is enhanced, which may offer some clues for the function of APP in cell survival [104]. Growth factor receptor-bound protein 2 (Grb2) is also a binding partner of the APP phosphorylated at Y682. Unlike proteins with PTB domains, Grb2 directly interacts with APP via the Src homology (SH) 2 region [66,105]. Zhou and colleagues discovered that the overexpression of the Grb2-SH2 domain elevates the production of Aβ40 in human embryonic kidney (HEK) 293 cells [66]. A recent study noted that the Y682 phosphorylation of APP is associated with the activation of the non-tyrosine kinase Fyn [69]. The increased phosphorylation at Y682 residues disrupts the binding of APP to clathrin and AP2 and its colocalization with these proteins [69]. In addition, the trafficking and sorting of APP after phosphorylation is also altered as more APP molecules accumulate in the trans-Golgi network (TGN) and late endosomes [69]. Therefore, the mistrafficking of APP due to Y682 phosphorylation might interfere with the cleavage of APP and the generation of Aβ in neurons. Through the use of an APP knock-in mouse model, it has been found in vivo that mutating the Y682 residue of APP to a non-phosphorylatable glycine (G) strongly enhances the α-secretase cleavage of APP in vivo [106]. The Y687 residue in the same motif has also been found to be phosphorylated in AD brains [38]. Rebelo et al. compared how the phosphorylation and dephosphorylation of Y687 residues affect the subcellular localization of APP. They discovered that Y687 phosphorylation significantly retains APP in the ER and TGN and reduces the generation of Aβ in neurons [70,71]. In contrast, APP dephosphorylated at Y687 has a higher turnover rate and is less enriched in the TGN than phosphorylated APP, and in turn increases the β-secretase cleavage of APP [70,71]. In accordance with these results, the mutation of the Y687 residue of APP to an alanine (A) or treatment with a tyrosine kinase inhibitor significantly reduces the cell surface localization of APP and the content of α-secretase cleavage products by deactivating the phosphorylation [107].

It is evident from these findings that the phosphorylation of APP at its cytoplasmic domain has a significant effect on its proteolytic cleavage and functions. These effects can be attributed directly to the modulation of trafficking and subcellular localization of APP after phosphorylation, or indirectly to the influence on the interaction between APP and its binding partners (Table 1). It is relatively clear that the phosphorylation of APP at T668 residues probably has a detrimental impact on AD pathology by promoting the amyloidogenic processing pathway. For the other phosphorylation sites, the outcome of kinase phosphorylation might depend on the regulation of signaling pathways and downstream effectors.

The phosphorylation of the extracellular domain of APP is rare, while the serine residues in the extracellular domain of APP can also be phosphorylated, as demonstrated by ^32^P isotope labeling experiments [108]. Two serine residues (S198 and S206) in the ectodomain have been reported to be phosphorylated under physiological conditions. Walter et al. found two distinct mechanisms of ectodomain phosphorylation of APP in neurons. In the first mechanism, the ectodomain of APP is phosphorylated by ectoprotein kinases in post-Golgi compartments such as secretory vehicles. However, mature APP proteins on the cell surface may also undergo phosphorylation at the extracellular domain by membrane-bound ectoprotein kinases [72]. Walter et al. further identified that casein kinase (CK)-1- and CK-2-like ectoprotein kinases are responsible for the ectodomain phosphorylation of membrane-anchored and secreted forms of APP [73], and that this process can be inhibited by heparin. This type of ectodomain phosphorylation might be important for APP trafficking and localization, as well as for its biological function [109].

### 3.2. α-Secretase Phosphorylation

The α-secretase mediated cleavage of APP is mainly performed by membrane bound proteases of the ADAM family. In particular, ADAM10 and ADAM17 are the major catalytic enzymes of this process [110,111]. ADAM10 has several possible phosphorylation sites in its cytoplasmic domain, but little research has reported on its phosphorylation. Saraceno and colleagues revealed that PKC is able to phosphorylate the S741 residue in the C-terminal domain of ADAM10 [112], however, the detailed function of this phosphorylation has not yet been defined. This phosphorylation does not interfere with the synapse-associated protein-97 (SAP97) mediated ADAM10 trafficking from the Golgi apparatus to the synaptic membrane [112]. The ADAM17 can not only process tumor necrosis factor α (TNF-α), but also participate in the cleavage of many other transmembrane proteins, such as TrkA and the cellular prion protein (PrPc). Díaz-Rodríguez et al. demonstrated in their study that the ERK phosphorylates ADAM17 at the T735 residue, leading to an accumulation of the truncated fragment of TrkA [113]. Further study on the subcellular localization of ADAM17 with or without phosphorylation has indicated that ADAM17 phosphorylated at T735 primarily colocalizes with TGN markers, but that unphosphorylated ADAM17 mainly resides in the ER [114]. Additionally, ERK activation can also induce the maturation of pro-TNF-α converting enzyme (pro-TACE) and the trafficking of TACE (ADAM17) to the cell surface [114]. This modulation may have an impact on the subsequent processing of membrane proteins that are substrates of ADAM members. For example, the physiological processing of PrPc can be increased by the upregulation of the phosphorylation and activity of ADAM17 following the activation of muscarinic receptors [115]. Nevertheless, the exact role of α-secretase phosphorylation in APP processing and Aβ generation remains to be investigated.

### 3.3. β-Secretase Phosphorylation

The aberrant activation of BACE1 has been documented both in normal aging and in AD [116,117]. In neurons in which BACE1 is the predominantly expressed form of β-secretase, the enzyme undergoes reinternalization and trafficking between the cell surface and endosomes. Walter et al. first reported that mature BACE1 can be phosphorylated by CK-1 at the S498 residue in its cytoplasmic domain [118]. The phosphorylation of BACE1 at the S498 residue regulates the retrieval of BACE from endocytosed vesicles and relocates the enzyme to juxtanuclear Golgi compartments, while the unphosphorylated BACE1 S498A mutant is retained in early endosomes [118]. The S498 phosphorylation can also influence the binding of BACE1 to some regulatory proteins such as the sorting adaptor Golgi-localized γ-ear-containing ARF-binding (GGA). The binding between BACE1 and GGA1 is mainly found in the TGN compartment and can be enhanced by S498 phosphorylation [119]. Afterwards, BACE1 molecules are delivered to the cell surface or to cell compartments where the APP cleavage occurs and may affect the generation of Aβ. Indeed, Sun et al. discovered a significant decrease in the S498 phosphorylation of BACE1 in AD patients [120]. Their study also proved that the polarity protein Par3 regulates the retrograde trafficking of BACE1 from the endosome to the TGN by atypical PKC (aPKC)-mediated S498 phosphorylation. The absence of Par3 reduces the S498 phosphorylation of BACE1, increases the convergence of APP and BACE1, and retains the enzyme in acidic compartments such as endosomes/lysosomes, in which the β-secretase cleavage of APP usually takes place [120,121]. CDK5 dysregulation has been implicated in AD. In addition to its role in phosphorylating APP, CDK5 is also capable of phosphorylating BACE1. Song and co-workers demonstrated that the level of phosphorylated BACE1 is elevated in brains of AD patients and might correlate with the increase in p25/CDK5 in the CNS. CDK5 phosphorylates BACE1 at the T252 residue in the lumen of endosomes and greatly increases the activity of BACE1, further leading to accelerated AD pathology [116].

### 3.4. γ-Secretase Phosphorylation

There are four subunits in the γ-secretase complex, each with distinct functions and regulatory pathways. PS is the catalytic subunit of γ-secretase and is rich of serine, threonine, and tyrosine residues, suggesting that it can be phosphorylated by kinases [122]. The phosphorylation of PS has a significant influence on the structure and function of the protein. Walter et al. demonstrated in 1996 that PS2 can be phosphorylated by CK-1 and CK-2 at serine residues in the N-terminus [123]. The C-terminal fragment of PS1 can be phosphorylated by PKC at serine residues, while this does not happen in the full-length PS1 [124]. The S353 and S357 residues of PS1 have been found to be phosphorylated by GSK-3β [125]. Upon phosphorylation, PS1 undergoes conformational changes that are not preferred for the binding to β-catenin [125]. The β-catenin molecules are stabilized, and their nuclear signaling is enhanced. Another study reported that GSK-3β mediated PS1 phosphorylation impairs the interactions among PS1, N-cadherin, and β-catenin, which are important for the cell surface expression and cellular signaling of PS1 [126]. Surprisingly, the phosphorylation of these two serine residues of PS1 by GSK-3β was found to increase the ratio of Aβ42/40 [127]. The phosphorylation of PS1 at the S397 site by GSK-3β can downregulate the level of the C-terminal fragment of PS1 by accelerating its turnover rate [128]. PKC, but not protein kinase A (PKA), phosphorylates the S346 residue of PS1, which is located in the caspase recognition motif. This phosphorylation can reduce the proteolytic processing of PS1 by caspases, and counteract the progression of apoptosis [129]. The dual-specificity tyrosine(Y)-phosphorylation-regulated kinase 1A (Dyrk1A) has been found to increase the production of Aβ in the brain. Ryu and co-workers revealed that Dyrk1A is able to phosphorylate PS1 at the T354 residues [130]. This phosphorylation can stabilize PS1 and increase the activity of γ-secretase, thereby stimulating the generation of Aβ [130]. Pro-inflammatory cytokines such as TNF-α are commonly present in AD patients [131]. TNF-α is able to activate JNK-dependent MAPK signaling cascades to induce the generation of Aβ [132]. Kuo et al. demonstrated that the phosphorylation of PS1 and nicastrin at the S319 and T320 residues stabilizes the C-terminal fragment of PS1 necessary for the catalytic ability of γ-secretase [133]. Nicastrin, the other subunit of γ-secretase, can also be phosphorylated by ERK1/2 [37]. Activated ERK1/2 can directly interact with and phosphorylate nicastrin (the detailed site is still unknown) in the complex, and downregulate the activity of γ-secretase complexes [134]. In general, there is less research on the phosphorylation of γ-secretase than that of other secretases, partly because of the complex structure of this enzyme.

## 4. Targeting the Phosphorylation Signaling in APP Processing for the Intervention in AD

As has been summarized before, the phosphorylation signaling in APP processing involves a variety of kinases, phosphorylation sites, and substrate proteins and has divergent effects on the function and proteolytic processing of APP. Since kinases are broadly involved in regulating different biological events, it might be challenging to specifically address the phosphorylation of proteins involved in APP metabolism in the brain without influencing other physiological functions of the kinase. The BBB poses additional challenges for drug candidates to enter the target brain regions [135]. Many kinase inhibitors have been developed for the treatment of cancer in the periphery, but their BBB penetrating properties and efficacy in the CNS are still in need of investigation [136]. Currently, there are some kinase inhibitors in clinical trials for neurological diseases, such as glioma, meningioma, and neurodegenerative disorders including AD and PD. Many small molecule-based kinase inhibitors are being tested in preclinical studies for their efficacies in neurodegeneration, brain tumors, and CNS injuries [136]. In the field of AD, a majority of kinase inhibitors, including CDK5 and GSK-3 inhibitors, have been developed to target tau hyperphosphorylation [137]. For example, the GSK-3 inhibitor lithium is undergoing clinical trials for patients with cognitive disorders [138]. It was reported that lithium treatment was able to improve the cognitive performance of amnestic MCI patients in a small-scale randomized control trial [138]. Surprisingly, long-term treatment with lithium seems to increase the Aβ42 level in the CSF [138]. Previous studies have established that lithium reduces the secretion of Aβ proteins by suppressing GSK-3α and 3β activities [139,140]. As GSK has been revealed to phosphorylate APP and regulate Aβ production as well as phosphorylated tau, it is possible that GSK mediated APP phosphorylation is also altered under lithium treatment. The convergence of Aβ pathology and tau pathology through protein kinases provides possibilities to overcome the key pathological features of AD simultaneously using one drug candidate [141]. AZD0530 is a Src tyrosine kinase inhibitor under investigation for sarcoma. It is believed that Aβ oligomers can interact with PrPc and activate the Fyn kinase, leading to synaptotoxicity and tau pathology [142]. van Dyck and co-workers tested the efficacy of AZD0530 in mild AD patients with increased Aβ levels. Although animal studies exhibited promising outcomes in the synaptic function and cognitive ability, the clinical trial did not show significant improvement in the symptoms of AD patients [143]. In fact, kinase inhibitors and phosphatase activators are commonly tested to investigate phosphorylation signaling pathways to corroborate the involvement of certain kinases in the regulation of APP processing, nevertheless this does not necessarily support the application of these compounds for the intervention of AD. However, there are still some therapeutics targeting the phosphorylation signaling of APP and secretases for the intervention of Aβ-related pathologies.

The phosphorylation of APP at T668 is believed to be an important clinical feature in brains of AD patients and has been recognized to accelerate Aβ generation and plaque formation. It might be beneficial to block this phosphorylation event in the brain in order to ameliorate AD pathology. The mutation of T668A in APP could prevent the phosphorylation of APP in vivo and rescue memory and synaptic plasticity deficits in a familial AD mouse model [144]. Insulin and insulin-like growth factor I (IGF-I) are now under clinical trial for AD [145]. Data from Kim et al. proved that both insulin and IGF-I can reduce the phosphorylation of APP at the T668 residue by activating the PI3K/Akt pathway [146]. The effect of insulin and IGF-I on APP phosphorylation is undermined by the overactivation of the PI3K/Akt pathway such as in the case of insulin resistance [146]. The NGF-TrkA pathway not only regulates the phosphorylation of Y682 but also has an impact on T668 phosphorylation. NGF can downregulate the p54 isoform of JNK and reduce the T668 phosphorylation of APP, enhancing the binding between APP and TrkA and the non-amyloidogenic APP processing [147]. DAPK1 is another emerging target for the modulation of APP phosphorylation at T668. Our previous studies showed that the activation of DAPK1 is related to APP phosphorylation and Aβ generation in AD patients [54]. C6 is a selective DAPK1 inhibitor with a high binding affinity for the kinase. This compound can significantly reduce the secretion of Aβ40 and Aβ42 and rescue AD pathology by suppressing the phosphorylation of APP at T668 in cell experiments and animal models, respectively [54,148,149]. It should be mentioned that DAPK1 also plays an important role in regulating cell apoptosis and tumor suppression [62]; therefore, a reasonable manipulation of DAPK1 activity should be considered to overcome the potential side effects of DAPK1 inhibition. Dyrk1 inhibition can synergistically inhibit APP phosphorylation at T668 and tau hyperphosphorylation, leading to significant delays in the onset of AD-like pathologies and improvement in the cognitive ability of mouse models [150,151]. Luteolin is a flavonoid compound derived from citrus. It can reduce the activity of GSK-3β and promote the phosphorylation of the C-terminal fragment of PS1, further disrupting the association between APP and PS1 and reducing the content of Aβ [152]. Using sodium selenite as an activator of ERK1/2, Tung and colleagues found that this compound decreases the activity of γ-secretase and the production of Aβ, probably by altering the ERK1/2 mediated phosphorylation of both nicastrin and PS1 [153]. A better understanding of how the phosphorylation signaling is regulated in APP processing would greatly aid in the development of selective and effective modulators. In addition, more animal studies are required to evaluate the efficacy and mechanisms of these compounds and kinase inhibitors on AD pathology prior to clinical research.

## 5. Concluding Remarks

AD is a complex neurodegenerative disease with a multifaceted pathogenesis. APP processing is a fundamental aspect both under physiological and pathological conditions. Although the enzymatic cleavage pathways of APP are relatively well-defined, the regulation of APP processing by different signaling pathways is not completely understood. Phosphorylation/dephosphorylation represent a key mechanism of the modulation of protein functions and structures. In AD, the phosphorylation of APP has been established at multiple levels, including both human and animal studies. The close link between the abnormal phosphorylation of proteins and AD pathology emphasizes that phosphorylation signaling pathways might be dysregulated during disease progression. Some of the phosphorylation sites, such as the T668 residue, can be phosphorylated by several kinases. Phosphorylation may directly influence the subcellular distribution of APP and therefore the proteolysis, or indirectly affect the binding between APP and other proteins and then induce further changes (Table 1). Understanding the mechanism of secretases phosphorylation is also important for gaining more insight into the physiological function of these proteins (Table 2). It is evident from Table 2 that phosphorylation is also capable of changing the enzymatic activity or the subcellular distribution of all three secretases in different ways. The direct inhibition of the catalytic ability of β- and γ-secretases has proved to be unsuitable for the prevention of AD, because of the important physiological functions of these secretases in processing other proteins. Therefore, selectively modulating the phosphorylation of secretases to minimize unwanted side effects while inhibiting the amyloidogenic pathway might offer new ideas for disease intervention. It is also interesting to observe that some of the kinases such as CDK5 and JNK can dually phosphorylate tau and APP proteins, suggesting that the malfunction of phosphorylation signaling might represent a common mechanism that drives Aβ and tau pathologies in AD. Moreover, these common signaling pathways and kinases may also explain the interplay between Aβ aggregation and tau tangle formation in the brain, and act as drug targets for the management of both pathological changes. In conclusion, it is of great value to elucidate the phosphorylation signaling in APP processing by identifying novel kinases and phosphorylation sites and designing safe and efficient modulators to gain an in-depth understanding of disease etiology and develop potential therapeutics.

## Figures and Tables

**Table 1:** Reported phosphorylation sites of APP695 and its effects.

Phosphorylation Site	Kinases/Signaling Pathways	Reported Effects	References
Threonine 654	CaMKII	N.D. ^1^	[55]
	ROCK2	Blocking T654 phosphorylation; promoting BACE1 relocation from endosomes to lysosomes and APP trafficking to lysosomes; reducing Aβ generation when inhibited	[56]
Serine 655	PKC, CaMKII	Increasing the secretion of APP into cerebrospinal fluid and decreasing the cleavage of mature APP; enhancing APP secretory traffic when activated	[55,57,58,59]
	ROCK1	Enhancing the interaction between BACE1 and APP and promoting Aβ generation when activated	[60]
	APP kinase I	Putatively modulating the internalization of APP when activated	[61]
Threonine 668	CDC2 kinase	Increasing the content of immature APP and C-terminal fragments while reducing the level of secreted APP products when activated	[40]
	CDK5	Enhancing the secretion of Aβ, sAPPβ, and sAPPα; enriching APP in endosomes when activated	[43,44,49]
	GSK-3β	Affecting copper-induced APP trafficking to axons	[41,42]
	JNK	Inducing APP degradation, lowering sAPPβ and Aβ generation, and promoting non-amyloidogenic processing when inhibited	[46,51,52]
	JIP-3-JNK	Phosphorylating APP and transporting pAPP to neuritis when activated	[50]
	DAPK1	Shifting APP toward non-amyloidogenic pathway and decreasing Aβ generation when inhibited	[54,62]
	LRRK2	Elevating the nuclear translocation of AICD and its transcriptional activity and exacerbating dopaminergic neurons when activated	[63]
	Plk2	Accelerating APP amyloidogenic cleavage by β-secretase at synapses when activated	[64]
Serine 675	Plk2	Stimulating the endocytosis of APP and driving the BACE1 cleavage; promoting meprin β mediated APP processing when activated	[64]
Tyrosine 682	Abl	Phosphorylating APP and forming stable complexes with pAPP; affecting APP binding to FE65 and X11 when activated	[65]
	Src	Increasing the formation of pAPP-Grb2 complexes when activated	[66]
	NGF-TrkA	Reducing the generation of the AICD; regulating the subcellular distribution and activation of TrkA when activated	[67,68]
	Fyn	Affecting the correct APP trafficking and sorting in neurons and the binding with clathrin and AP2 when activated	[69]
Tyrosine 687	Tyrosine kinase	Retaining APP in ER and TGN and decreasing its turnover rate; reducing Aβ formation when activated	[70,71]
Tyrosine 653	N.D.	N.D.	[38]
Threonine 686	N.D.	N.D.	[38]
Serine 198 and serine 206	CK-1 and CK-2-like ectoprotein kinases	Essential for the correct location of APP on the cell surface	[72,73]

Abbreviations: ^1^ N.D: not defined in the literature; CaMKII: Ca^2+^/calmodulin-dependent protein kinase II; ROCK2: Rho-associated coiled-coil kinase 2; BACE1: β-site APP-cleaving enzyme 1; APP: amyloid precursor protein; Aβ: amyloid-β; PKC: protein kinase C; ROCK1: Rho-associated coiled-coil kinase 1; CDC2: p34cdc2 protein kinase; CDK5: cyclin-dependent kinase 5; sAPPβ; soluble APPβ; sAPPα: soluble APPα; GSK-3β: glycogen synthesis kinase-3β; JNK: c-Jun N-terminal kinase; JIP-3: JNK-interacting protein 3; DAPK1: death-associated protein kinase 1; LRRK2: leucine-rich repeat kinase 2; AICD: APP intracellular domain; NGF: nerve growth factor; ER: endoplasmic reticulum; TGN: trans-Golgi network; CK: casein kinase.

**Table 2:** Reported phosphorylation sites of α- and β-secretases and subunits of γ-secretase and their effects.

Secretase or Subunit	Phosphorylation Site	Kinases/Signaling Pathways	Reported Effects	References
ADAM10	Serine 741	PKC	No effect on the interaction between ADAM10 and SAP97	[112]
ADAM17	Threonine 735	ERK	Inducing the maturation of pro-TACE protein and the trafficking of TACE to the cell surface when activated	[113,114]
BACE1	Serine 498	CK-1	Transferring BACE1 to juxtanuclear Golgi compartments when activated	[118]
	Serine 498	N.D. ^1^	Affecting the binding between GGA1 and BACE1	[119]
	Serine 498	aPKC	Increasing the convergence of APP and BACE1 and retaining the enzyme in acidic compartments when activated	[120]
	Threonine 252	p25-CDK5	Increasing the activity of BACE1 when activated	[116]
PS1	Serine 353 and serine 357	GSK-3β	Disrupting the interaction between PS1, β-catenin, and N-cadherin; increasing the Aβ42/40 ratio when activated	[125,126,127]
	Serine 397	GSK-3β	Increasing the level of PS1 C-terminal fragments when activated	[128]
	Serine 346	PKC	Reducing the proteolytic processing of PS1 by caspases when activated	[129]
	Threonine 354	Dyrk1A	Stabilizing PS1 and increasing the activity of γ-secretase; stimulating the generation of Aβ when activated	[130]
	Serine 319 and Threonine 320	JNK	Enhancing the stability of the PS1 C-terminal fragment when activated	[133]
Nicastrin	N.D.	ERK1/2	Downregulating the activity of γ-secretase complexes when activated	[134]

Abbreviations: ^1^ N.D.: not defined in the literature; ADAM: a disintegrin and metalloproteinase; ERK: extracellular signal-regulated kinase; TACE: TNF-α converting enzyme; GGA1: Golgi-localized γ-ear-containing ARF-binding protein 1.
